# Anomalies de l'hémogramme dans l'association drépanocytose et paludisme chez l'adulte en hématologie clinique au CNHU-HKM de Cotonou (Bénin)

**DOI:** 10.48327/mtsi.v4i1.2024.404

**Published:** 2024-01-17

**Authors:** Alban Gildas Comlan ZOHOUN, Tatiana BAGLOAGBODANDE, Axel ADANHO, Romaric MASSI, Bienvenu HOUSSOU, Gnon Gourou OROU GUIWA, Justin DÈHOUMON, Josiane MEHOU, Ludovic ANANI, Anne VOVOR, Dorothée KINDEGAZARD

**Affiliations:** 1 Laboratoire d'hématologie, Clinique universitaire des maladies du sang, Centre national hospitalier universitaire Hubert Koutoukou Maga (CNHU-HKM), Cotonou, Bénin; 2 Faculté des sciences de la santé, Université d'Abomey-Calavi, Cotonou, Bénin; 3 Faculté des sciences de la santé, Université de Lomé, Togo Auteur

**Keywords:** Hémogramme, Paludisme, Drépanocytose, Bénin, Afrique subsaharienne, Blood count, Malaria, Sickle cell disease, Benin, Sub-Saharan Africa

## Abstract

**Introduction:**

Bien qu'un effet protecteur de l'hémoglobine S ait été décrit, le paludisme a fréquemment été associé à l'augmentation de la morbi-mortalité des malades drépanocytaires en Afrique. Diverses cytopénies sont fréquentes et retrouvées sur l'hémogramme de ces patients. Ce travail avait pour objectif, en zone d'endémie palustre et de forte prévalence de la drépanocytose qu'est le Bénin, d’établir et de comparer le profil de l'hémogramme selon le type hémoglobinique dans l'association drépanocytose et paludisme.

**Matériel et méthode:**

Une étude prospective à visée descriptive a été réalisée d'octobre 2020 à octobre 2022. Elle a inclus tous les patients atteints de syndrome drépanocytaire majeur, chez qui une goutte épaisse avec calcul de la densité parasitaire (GEDP) était réalisée. La goutte épaisse était considérée comme positive, quelle que soit la valeur de la parasitémie. Pour chaque patient, un hémogramme a été réalisé sur l'automate Sysmex XT 4000i complété par une étude de frottis après coloration au May-Grünwald Giemsa. Les données ont été analysées à l'aide du logiciel R 3.6.1.

**Résultats:**

Trois cents cas non redondants avec goutte épaisse positive ont été recensés chez les patients drépanocytaires dont 208 homozygotes SS (69,3 %) et 92 hétérozygotes SC (30,7 %) contre 181 cas non redondants avec une goutte épaisse négative dont 119 homozygotes SS (65,7 %) et 62 hétérozygotes SC (34,3 %). Pour les sujets avec une GEDP positive, la majorité des patients (70 %) présentait une symptomatologie fonctionnelle ou physique. Le paludisme grave concernait 58 % des cas recensés. La proportion de paludisme grave était plus élevée chez les patients homozygotes SS que chez les doubles hétérozygotes SC (p < 0,0001). La densité parasitaire moyenne était plus élevée chez les sujets SS (4 320,7 ± 2 185 trophozoïtes/pl) que chez les sujets SC (1 564,4 ± 1 221 trophozoïtes/pl; p <0,0001). *Plasmodium falciparum* a été la seule espèce identifiée. Le taux d'hémoglobine moyen des sujets SS impaludés était de 6,1 g/dl et significativement inférieur à celui retrouvé chez les sujets SS non impaludés (p < 0,0001). La leucocytose moyenne des sujets SS impaludés était de 16,58 G/l contre 13,2 G/l chez ceux avec une goutte épaisse négative (p < 0,0001). Vingt cas de thrombopénie ont été retrouvés chez les sujets SS avec une goutte épaisse positive contre 6 cas chez ceux avec une goutte épaisse négative. En ce qui concerne les sujets SC avec une goutte épaisse positive, les valeurs moyennes du taux d'hémoglobine et du nombre de leucocytes étaient respectivement de 9,8 g/dl et 10,63 G/l contre 11,27 g/dl et 7,3 G/l pour les sujets SC avec une goutte épaisse négative. Dix-huit cas de thrombopénie ont été retrouvés chez les sujets avec une goutte épaisse positive contre 17 cas chez ceux avec une goutte épaisse négative.

**Conclusion:**

En Afrique subsaharienne, l'hémogramme constitue l'examen le plus accessible aux populations notamment les plus démunies. Chez le sujet drépanocytaire, l’étude et la connaissance des anomalies de l'hémogramme au cours du paludisme mettent à la disposition des cliniciens des outils de prise de décision diagnostique et thérapeutique.

## Abstract (en)

**Introduction:**

Although a protective effect of hemoglobin S has been described, malaria has frequently been associated with increased morbidity and mortality in sickle cell disease patients in Africa. Various cytopenias are frequently found on the haemograms of these patients. In Benin, a malaria-endemic zone with a high prevalence of sickle cell disease, the aim of this study was to establish and compare the blood count profile according to hemoglobin type in the association of sickle cell disease and malaria.

**Material and method:**

This was a prospective descriptive study. It covered a 24-month period from October 2020 to October 2022. It included all patients with major sickle cell syndrome seen in clinical haematology and with a positive thick drop/parasite density, whatever the parasitaemia value. For each patient, a blood count was performed on the Sysmex XT 4000i machine, supplemented by a smear study after staining with May-Grunwald Giemsa. Data were analyzed using R 3.6.1 software.

**Results:**

Three hundred non-redundant cases with a positive thick smear were identified in sickle cell patients, including 208 SS homozygotes (69.3%) and 92 SC heterozygotes (30.7%). In contrast, there were 181 non-redundant cases with a negative thick smear, including 119 SS homozygotes (65.7%) and 62 SC heterozygotes (34.3%). Among subjects with a positive thick smear, the majority of patients (70%) exhibited clinical symptoms. Severe malaria was observed in 58% of the cases. The proportion of severe malaria was higher in SS homozygote patients than in double heterozygote SC patients (p < 0.0001). The mean parasite density was higher in SS individuals (4 320.7 ± 2 185 trophozoites/pL) compared to SC individuals (1 564.4 ± 1 221 trophozoites/pL; p < 0.0001). *Plasmodium falciparum* was the only species identified. The mean hemoglobin level in impaludated SS subjects was 6.1 g/dL, significantly lower than that in non-impaludated SS subjects (p < 0.0001). The average white blood cell count in impaludated SS subjects was 16.58 G/L, compared to 13.2 G/L in those with a negative thick smear (p < 0.0001). Twenty cases of thrombocytopenia were found in SS subjects with a positive thick smear, compared to 6 cases in those with a negative thick smear. As for SC subjects with a positive thick smear, the average hemoglobin levels and white blood cell counts were 9.8 g/dL and 10.63 G/L, respectively, compared to 11.27 g/dL and 7.3 G/L in SC subjects with a negative thick smear. Eighteen cases of thrombocytopenia were found in subjects with a positive thick smear, compared to 17 cases in those with a negative thick smear.

**Discussion:**

Sickle cell disease and malaria represent two major public health problems. However, contrary to popular belief, sickle cell disease is not immune to malaria infestation. Malaria is recognized as one of the main causes of morbidity and mortality in sickle cell patients, particularly children. In Benin, its association with sickle cell emergencies has already been reported.

Our study found that malaria was predominantly associated with the homozygous SS form (p < 0.00001). Severe malaria was the most common clinical form. All malaria infestations in our series were due to *Plasmodium falciparum,* and parasitaemia was significantly higher in SS patients (p < 0.0001).

The hematological profile of the association of sickle cell disease and malaria in homozygous SS individuals in our series showed characteristics of a normocytic normochromic anemia with neutrophil-predominant leukocytosis. Compared to non-malaria-infected SS individuals, there was a significant worsening of anemia, neutrophil-predominant leukocytosis, and a decrease in the average platelet count. In SC individuals, there was rather a microcytic normochromic regenerative anemia associated with neutrophil-predominant leukocytosis. Compared to non-malaria-infected SC individuals, there was a significant decrease in the rate of anemia and neutrophil-predominant leukocytosis. Anemia is a constant feature in homozygous sickle cell disease, and the low values recorded illustrate the hemolytic nature of malaria, especially in SS individuals, and the better tolerance of SC individuals. Furthermore, the low baseline hemoglobin levels make SS individuals more vulnerable to malaria-induced anemia compared to SC individuals. The observed leukocytosis is generally accompanied by reticulocytosis in the case of major sickle cell syndrome, which must be taken into account for result validation. It is the expression of compensatory bone marrow reaction to anemia and inflammatory mechanisms resulting from malaria infestation. Finally, thrombocytopenia was significantly more common in SC patients, even though they were adults living in malaria-endemic areas. Malaria can frequently induce thrombocytopenia through platelet consumption during the “rosetting” phenomenon. In SS patients, the effects of “rosetting” could be compensated for by the bone marrow stimulation induced by anemia. In our series with adult subjects living in an endemic area, thrombocytopenia is not a frequent biological disturbance. In a clinicalbiological context combining a systemic inflammatory response syndrome with anemia and neutrophil-predominant leukocytosis in a SS or SC sickle cell patient, the clinician should be able to consider malaria and confirm or rule out this diagnosis.

## Introduction

La drépanocytose et le paludisme représentent deux problèmes majeurs de santé publique [10, 16]. Il existe une superposition entre les aires géographiques du paludisme et de la drépanocytose. Cette relation s'expliquerait par un avantage sélectif des hétérozygotes en zone d'endémie palustre et les mutations de la chaîne bêta de l'hémoglobine qui conféreraient un avantage contre le paludisme [6, 9, 23, 26]. Les mécanismes évoqués sont une inhibition de la croissance parasitaire et une destruction accélérée des hématies parasitées par la rate selon des processus immunologiques innés ou acquis [6, 8, 23]. Cependant, contrairement à l'idée véhiculée, la drépanocytose n'empêche pas les infestations palustres. Ainsi, le paludisme à *Plasmodium falciparum* est reconnu comme l'une des principales causes de morbimortalité chez les sujets drépanocytaires, notamment les enfants [25]. Au Bénin, le paludisme était retrouvé chez 12,5 % des sujets drépanocytaires dans la série de Rahimy *et al.* [21]. De même, Dodo *et al.* le retrouvaient associé à une urgence drépanocytaire chez 27,5 % des malades hospitalisés dans le service d'hématologie du Centre national hospitalier universitaire Hubert Koutoukou Maga (CNHU-HKM) de Cotonou [3]. Dans cette étude, notre objectif est de documenter les anomalies de l'hémogramme dans l'association paludisme et drépanocytose chez l'adulte au CNHU-HKM.

## Cadre et méthode d’étude

Il s'est agi d'une étude prospective à visée descriptive menée sur une période de 2 ans d'octobre 2020 à octobre 2022. Ont été inclus tous les patients drépanocytaires adultes SS ou SC, vus en hématologie clinique au CNHU-HKM de Cotonou et chez qui une goutte épaisse/densité parasitaire (GEDP) était réalisée.

La goutte épaisse était effectuée dans le service de Parasitologie-mycologie du même centre. La goutte épaisse et le frottis sanguin ont été confectionnés à partir du sang prélevé sur tube éthylène diamine tétra acétique (EDTA) selon la procédure standardisée du service.

Pour toute goutte épaisse positive, la densité parasitaire a été calculée en comptant le nombre de leucocytes et de parasites tandis que le frottis sanguin a permis l'identification de l'espèce de *Plasmodium* en cause. Les critères de gravité du paludisme utilisés sont ceux définis par l'Organisation mondiale de la Santé [18].

Les paramètres hématologiques ont été déterminés sur l'automate Sysmex XT 4000i dont le principe est basé sur le système d'hydro-focalisation et de la cytométrie en flux. Différentes méthodes sont utilisées pour les différents paramètres de l'hémogramme, tels que la spectrophotométrie pour le dosage de l'hémoglobine; l'impédancemétrie pour la numération des hématies, des leucocytes et des plaquettes ainsi que la fluorocytométrie pour la formule leucocytaire, la numération des réticulocytes et des érythroblastes. Les différents paramètres de l'hémogramme qui ont été déterminés étaient : le taux d'hémoglobine, l'hématocrite, le volume globulaire moyen, la concentration corpusculaire moyenne en hémoglobine, la teneur corpusculaire moyenne en hémoglobine, le nombre de plaquettes, le nombre de leucocytes et la formule leucocytaire permettant d'identifier le nombre de polynucléaires neutrophiles, de lymphocytes, de polynucléaires éosinophiles, de polynucléaires basophiles et le nombre de monocytes. Les contrôles de qualité interne de l'hémogramme ont été réalisés de façon journalière et les résultats ont fait l'objet d'une validation technique et biologique. La lecture de frottis sanguins était de réalisation systématique après fixation et coloration au May-Grünwald Giemsa devant toute anomalie de la formule automatisée.

Sur le plan éthique, le consentement libre et éclairé des patients inclus avait été obtenu. Les données ont été recueillies et traitées dans le strict respect de l'anonymat et de la confidentialité. Enfin, un avis éthique a été obtenu et enregistré sous le numéro REF 0397/CLERB-UP/P/SP/R/SA.

Les données ont été saisies et traitées par les logiciels Excel 2016 et ont été analysées et comparées entre elles à l'aide du logiciel R 3.6.1. Les moyennes de chaque paramètre ont été présentées avec les écarts-types en considérant un intervalle de confiance de 95 %. Le test de l’écart réduit a été utilisé pour comparer les moyennes et le seuil de signification a été fixé à p < 0,05.

## Résultats

Nous avons colligé 300 cas de paludisme non redondants chez les patients drépanocytaires dont 208 homozygotes SS (69,3 %) et 92 hétérozygotes SC (30,7 %) contre 181 patients drépanocytaires non redondants avec une goutte épaisse négative dont 119 homozygotes SS (65,7 %) et 62 hétérozygotes SC (34,3 %). Pour la population drépanocytaire avec la goutte épaisse/densité parasitaire positive, les patients de sexe masculin étaient au nombre de 169 (56,3 %) pour 131 sujets de sexe féminin (43,7 %). Le sex-ratio H/F était de 1,3. La moyenne d’âge de notre échantillon était de 26,6 ans avec des extrêmes de 18 et 70 ans. Rapportée au type hémoglobinique, la moyenne d’âge des sujets SS était inférieure à celle des sujets SC (24,8 ans contre 27,9 ans). La majorité des cas recrutés, 210 (70 %) ont été admis avec des signes fonctionnels et/ou physiques contre 90 (30 %) qui ont été vus en phase stationnaire lors d'une consultation de routine dans le cadre du suivi systématique. Pour la population drépanocytaire avec la goutte épaisse/densité parasitaire négative, la répartition par sexe a montré une prédominance féminine à 56,36 % correspondant à un sex-ratio H/F de 0,8. La moyenne d’âge au cours de notre étude était de 32 ans avec des extrêmes de 18 et 95 ans. La moyenne d’âge des sujets SS était de 29 ans contre 37 ans pour les patients SC.

Le Tableau I présente la répartition des échantillons GEDP positive et GEDP négative en fonction du sexe et du profil hémoglobinique.

**Tableau I:** Répartition des échantillons GEDP positive et GEDP négative en fonction du sexe et du profil hémoglobinique Distribution of positive thick smear parasite density and negative thick smear parasite density samples based on gender and hemoglobin profile

	GEDP positive				GEDP négative			
	SS		SC		SS		SC	
	n	%	n	%	n	%	n	%
**Masculin**	122	58,7	47	51,1	70	58,8	32	51,6
**Féminin**	86	41,3	45	48,9	49	41,2	30	48,4
**Total**	208	100	92	100	119	100	62	100

Concernant la description de la population GEDP positive avec une symptomatologie fonctionnelle et/ou physique, 162/210, soit 77.1 % étaient des homozygotes SS. De même, ceux vus en consultation de suivi étaient en majorité des homozygotes SS (55/90 soit 61.1 %). Le paludisme grave concernait 58 % des cas recensés. Les critères de définition de gravité étaient majoritairement biologiques et représentés dans le Tableau II.

**Tableau II:** Fréquence des signes de gravité biologiques du paludisme selon le phénotype de l'hémoglobine Frequency of severe malaria biological signs according to haemoglobin phenotype

Items	Sujets SC	Sujets SS	Total	P
	n (%)	n (%)	n	
**Anémie sévère (Taux d'hémoglobine < 7 g/dl)**	04 (4,1)	93 (95,9)	97	< 0,0001
**Hémoglobinurie**	07 (9,3)	68 (90,7)	75	0,1350
**Hyperbilirubinémie**	09 (13,2)	59 (86,8)	68	0,1350

La densité parasitaire moyenne était significativement plus élevée chez les homozygotes SS (4 320,7 ± 2 185 trophozoïtes/pl) que chez les hétérozygotes SC (1 564,4 ± 1 221 trophozoïtes/pl; p < 0,0001). *Plasmodium falciparum* était la seule espèce identifiée. Les gamétocytes étaient retrouvés dans 9 % des frottis. Ils étaient plus fréquemment observés chez les patients SS que chez les patients SC mais cette différence n’était pas statistiquement significative (p = 0,071). Les quatre frottis contenant des schizontes n’étaient observés que chez les malades SS.

Le taux d'hémoglobine moyen des sujets SS impaludés était de 6,3 g/dl et significativement inférieur à celui des sujets SS non impaludés qui était de 7,7 g/dl (p < 0,0001). Le taux d'hématocrite, le nombre de leucocytes, de polynucléaires neutrophiles et de plaquettes étaient significativement plus bas chez les sujets SS impaludés comparés à ceux non impaludés (p < 0,0001). Pour les sujets doubles hétérozygotes SC, ceux impaludés avaient des valeurs significativement inférieures à celles des sujets non impaludés (p < 0,000). Les paramètres de l'hémogramme concernés étaient représentés par : le taux d'hémoglobine, l'hématocrite, le volume globulaire moyen, le nombre de leucocytes, de polynucléaires neutrophiles, de polynucléaires éosinophiles et de monocytes. Concernant la fréquence de survenue de la thrombopénie dans le paludisme, 20 cas de thrombopénie avaient été retrouvés chez les sujets SS avec goutte épaisse positive contre 6 cas chez ceux avec une goutte épaisse négative. Le constat était différent chez les doubles hétérozygotes SC avec 18 et 17 cas de thrombopénie retrouvés respectivement chez les sujets avec goutte épaisse positive et ceux avec goutte épaisse négative.

L’étude comparative des anomalies des hémogrammes selon le type hémoglobinique et l'infestation palustre est résumée dans le Tableau III.

**Tableau III:** Comparaison des valeurs moyennes des principaux paramètres de l'hémogramme selon le phénotype de l'hémoglobine et l'infestation palustre Comparison of mean values of main haemogram parameters according to haemoglobin phenotype and malaria infestation

Paramètres	Sujets SS			Sujets SC		
	GEDP -	GEDP +	p	GEDP -	GEDP +	p
**Hémoglobine (g/dl)**	7,70	6,3	< 0,0001	11,27	9,8	< 0,0001
**Hématocrite (%)**	23	19,19	< 0,0001	33	28,33	< 0,0001
**VGM (fL)**	84	85,09	0,392	72	74,90	0,002
**TCMH (pg)**	28,5	27,96	0,281	25,5	25,94	0,324
**Plaquettes (G/l)**	426	307	< 0,0001	223	203	0,161
**Leucocytes (G/l)**	13,2	16,58	< 0,0001	7,3	10,63	< 0,0001
**Neutrophiles (G/l)**	6,93	10,05	< 0,0001	3,96	6,82	< 0,0001
**Éosinophiles (G/l)**	0,35	0,175	0,060	0,17	0,06	0,020
**Basophiles (G/l)**	0,04	0,035	0,924	0,04	0,02	0,550
**Monocytes (G/l)**	0,71	0,89	0,136	0,37	0,59	0,005
**Lymphocytes (G/l)**	4,94	5,10	0,654	2,62	2,92	0,231

VGM : volume globulaire moyen; TCMH : teneur corpusculaire moyenne en hémoglobine; GEDP - : goutte épaisse/densité parasitaire négative; GEDP + : goutte épaisse/densité parasitaire positive.

## Discussion

L'association paludisme et drépanocytose était plus fréquente dans la forme homozygote SS comparée à la forme hétérozygote SC comme rapporté dans la littérature [1, 2, 17, 24]. Le sexe ne semble pas influencer la survenue du paludisme dans le syndrome drépanocytaire majeur [12, 15, 20]. Les données de la littérature précisent que 90 % des enfants drépanocytaires homozygotes SS en Afrique subsaharienne meurent avant l’âge de 5 ans; toutefois la prise en charge précoce et le suivi des malades drépanocytaires dépistés avant 5 ans expliquent l'amélioration considérable de la survie drépanocytaire [11, 19, 22]. Il a été démontré une plus grande vulnérabilité du jeune adulte drépanocytaire en cas du relâchement des mesures de prophylaxie [7]. Le paludisme est non seulement une réalité chez le sujet drépanocytaire mais il survient principalement sous sa forme grave et affecte significativement les patients homozygotes SS. Le risque plus faible de paludisme grave chez les sujets SC est connu et en lien avec le polymorphisme équilibré de la mutation à l'origine du caractère protecteur de la mutation dans l'hétérozygotie alors qu'elle est délétère dans l'homozygotie [13]. La totalité des infestations palustres dans notre série était due à *Plasmodium falciparum* et se justifie par la forte prévalence de *P. falciparum* au Bénin. La densité parasitaire significativement plus faible chez les sujets SC pourrait s'expliquer par la structure particulière des récepteurs *PfEMP-1* des hématies contenant l'hémoglobine C qui limite l'adhérence de *P. falciparum* [4, 5].

En Afrique subsaharienne en général et au Bénin en particulier, l'accès aux soins de santé demeure limitée par les conditions socio-économiques moindres et l'absence de sécurité sociale. Pour les patients drépanocytaires, l'hémogramme demeure l'un des examens biologiques accessibles avec un coût variant selon les centres de santé de 5 000 à 10 000 francs CFA (soit 7,6 à 15,2 euros). Cet examen constitue également une mine d'indications pour le clinicien dans un but diagnostique et thérapeutique.

Le profil de l'hémogramme de l'association drépanocytose et paludisme chez le sujet homozygote SS dans notre série était celui d'une anémie normocytaire normochrome avec une hyperleucocytose à prédominance neutrophile. Comparé aux sujets SS non impaludés, on note de façon significative une aggravation de l'anémie, une hyperleucocytose à prédominance neutrophile et une diminution du nombre moyen de plaquettes. Chez les sujets SC, on observait plutôt une anémie microcytaire normochrome régénérative associée à une hyperleucocytose à prédominance neutrophile. En comparant aux sujets SC non impaludés, on note de façon significative une diminution du taux d'anémie et une hyperleucocytose à prédominance neutrophile. L'anémie est une constante dans la drépanocytose homozygote et les valeurs basses enregistrées illustrent le caractère hémolytique du paludisme notamment chez les sujets SS et la meilleure tolérance des sujets SC. De plus, les faibles valeurs basales du taux d'hémoglobine rendent les sujets SS plus vulnérables à l'anémie induite par le paludisme, par rapport aux sujets SC. L'hyperleucocytose observée s'accompagne généralement d'une réticulocytose en cas de syndrome drépanocytaire majeur dont il faudra tenir compte pour la validation des résultats. Elle est l'expression de la réaction médullaire compensatoire de l'anémie et des mécanismes inflammatoires résultant de l'infestation palustre. Le paludisme peut fréquemment induire une thrombopénie par consommation de plaquettes lors du phénomène de « rosetting » [14].

Chez les patients SS, les effets du « rosetting » pourraient être compensés par la stimulation médullaire induite par l'anémie. Dans notre série avec des sujets adultes vivants en zone d'endémie, la thrombopénie n'est pas une perturbation biologique fréquente.

Dans un contexte clinico-biologique associant un syndrome de réponse inflammatoire systémique à une anémie et une hyperleucocytose à prédominance neutrophile chez un patient drépanocytaire SS ou SC, le clinicien doit savoir évoquer un paludisme et confirmer ou infirmer ce diagnostic.

## Conclusion

Notre étude est la toute première au Bénin à s'intéresser aux variations de l'hémogramme dans le paludisme chez le patient drépanocytaire adulte béninois. Elle confirme la réalité du paludisme chez le drépanocytaire. Les paramètres hématologiques sont fonction du phénotype hémoglobinique. Nos résultats démontrent que les valeurs critiques sont l'apanage du sujet SS tandis que les valeurs sont améliorées chez le sujet SC. Une étude longitudinale portant sur une plus large cohorte permettra d'obtenir des données hémato-biologiques précises dans l'association drépanocytose paludisme au Bénin.

## Contribution des auteurs

A. ZOHOUN : conception de l’étude, recherche bibliographique, supervision de l’étude, validation et analyse des données, rédaction du manuscrit.

T. BAGLO-AGBODANDE : validation du protocole, validation et analyse des données, relecture du manuscrit.

A. ADANHO : recueil des données, recherche bibliographique et rédaction du manuscrit. R. MASSI, B. HOUSSOU, G. G. OROU GUIWA, J. DÈHOUMON, J. MEHOU : relecture du manuscrit.

L. ANANI, A. VOVOR et D. KINDE-GAZARD : validation du protocole, relecture et validation du manuscrit.

Tous les auteurs ont lu et approuvé la version finale du manuscrit.

## Liens d'intérêt

Les auteurs ne déclarent aucun lien d'intérêt.
